# PROMISE: Prognostic Radiomic Outcome Measurement in Acute Subdural Hematoma Evacuation Post-Craniotomy

**DOI:** 10.3390/brainsci15010058

**Published:** 2025-01-10

**Authors:** Alexandru Guranda, Antonia Richter, Johannes Wach, Erdem Güresir, Martin Vychopen

**Affiliations:** Department of Neurosurgery, University Hospital Leipzig, 04103 Leipzig, Germany; ar32june@studserv.uni-leipzig.de (A.R.); johannes.wach@uniklinik-leipzig.de (J.W.); erdem.gueresir@medizin.uni-leipzig.de (E.G.); martin.vychopen@uniklinik-leipzig.de (M.V.)

**Keywords:** acute subdural hematoma, surgical outcome, surface area, craniotomy, Feret diameter, 3D Slicer, radiomic

## Abstract

**Background/Objectives:** Traumatic acute subdural hematoma (aSDH) often requires surgical intervention, such as craniotomy, to relieve mass lesions and pressure. The extent of hematoma evacuation significantly impacts patient outcomes. This study utilizes 3D Slicer software to analyse post-craniotomy hematoma volume changes and evaluate their prognostic significance in aSDH patients. **Methods:** Among 178 adult patients diagnosed with aSDH from January 2015 to December 2022, 64 underwent hematoma evacuation via craniotomy. Initial scans were performed within 24 h of trauma, followed by routine postoperative scans to assess residual hematoma. We conducted radiomic analysis of preoperative and postoperative volumes, surface area, Feret diameter, sphericity, flatness, and elongation. Clinical parameters, including SOFA score, APACHE score, pupillary response, comorbidities, age, anticoagulation status, and preoperative haematocrit and haemoglobin levels, were also evaluated. **Results:** Changes in Δ surface area significantly correlated with 30-day outcomes (*p* = 0.03) and showed moderate predictive accuracy (AUC = 0.65). Patients with a Δ surface area > 30,090 mm^2^ experienced poorer outcomes (OR = 6.66, *p* = 0.02). Significant features included preoperative surface area (*p* = 0.009), Feret diameter (*p* = 0.0012). In multivariate analysis, only the Feret diameter remained significant (*p* = 0.01). **Conclusions:** Postoperative Δ surface area is, among other variables, a strong predictor of 30-day outcomes, while in multivariate analysis, preoperative Feret diameter remains the only independent predictor. Radiomic analysis with 3D Slicer may enhance prognostic accuracy and inform tailored therapeutic strategies.

## 1. Introduction

Acute subdural hematoma (aSDH) is a life-threatening neurosurgical condition characterized by the accumulation of blood in a concave shape between the dural and arachnoid meningeal layers, typically following traumatic brain injury (TBI). This blood collection can cause intracranial hypertension and potentially result in brain herniation if not treated promptly. Mortality rates range from 40% to 60%, particularly among older adults with increasing numbers of comorbidities [1,2,3]. The incidence of aSDH has been increasing with age over recent decades [4].

In instances where the patient’s condition stabilizes, long-term management strategies, such as chronification, may be considered therapy of choice [5]. However, if clinical deterioration occurs, craniotomy and hematoma evacuation remain the only solution to reduce the mass effect of the clot and prevent cerebral herniation [6,7]. Postoperative outcomes can vary significantly based on factors including patient age, preexisting medical conditions, the severity of the brain injury, and the efficacy of the hematoma evacuation [8]. Common comorbidities associated with aSDH include cardiac abnormalities [9], chronic renal insufficiency requiring haemodialysis [10], and the use of antithrombotic therapy [11]. All these factors are reported to influence both short- and long-term outcomes.

Conventional predictors of outcomes in acute subdural hematoma include clinical parameters such as Glasgow Coma Scale (GCS) score [12], pupillary abnormalities [13], and hematoma volume [14]. Haematocrit may also play a role in managing aSDH [15]. Additionally, haemoglobin levels may impact aSDH outcomes, with low levels impairing oxygen delivery. Anaemia might contribute to the development of secondary injury in patients with acute traumatic brain injury [16].

While these metrics offer valuable prognostic information, they fail to fully account for the variability in patient outcomes, underscoring the necessity for more advanced prognostic tools.

Recently, radiomics has emerged as a promising tool for predicting outcomes in various medical areas, like oncology [17], neurology [18], and traumatic brain injuries [19]. Essentially, radiomics involves extracting detailed quantitative measurements from medical images to identify patterns that might present a useful prognostication tool. These measurements—encompassing features such as shape, texture, and intensity—may reveal information not apparent through expert evaluation alone and could contribute to the development of more advanced prognostic models [20].

In the context of aSDH, applying radiomics to analyse computed tomography (CT) scans may facilitate both qualitative and quantitative assessments of the hematoma, aiding in outcome prediction post-surgery and informing postoperative care.

This study aims to utilize radiomics to explore how changes in hematoma volume and shape after craniotomy might serve as predictors of patient outcomes in cases of acute subdural hematoma, alongside already known effects of clinical and laboratory parameters.

## 2. Materials and Methods

### 2.1. Study Design and Population

This study is a retrospective analysis conducted at the University Hospital Leipzig, focusing on patients diagnosed with aSDH between January 2015 and December 2022. The study protocol was reviewed and approved by the Clinical Ethics Committee of the University of Leipzig (362/23-ek).

A total of 178 adult patients with confirmed aSDH were initially identified from our institutional database. The inclusion criteria were as follows: (1) diagnosis of aSDH confirmed by CT, (2) performance of craniotomy for hematoma evacuation, and (3) availability of both preoperative and postoperative CT scans acquired within 24 h following the head injury. Exclusion criteria included patients younger than 18 years and those with incomplete imaging or clinical data.

Of these, only the 64 patients who underwent craniotomy were included, as the study specifically focuses on radiomic outcomes following this procedure. Patients treated with hemicraniectomy, burr hole trepanation, or conservative management were excluded due to differences in surgical techniques and imaging characteristics, which would impede comparability in radiomic analysis.

The diagnosis of aSDH was based on cranial CT findings, defined as a hyperdense, concave lesion adjacent to the brain parenchyma, not limited by cranial sutures, and typically extending over a broad cortical area [21]. All cases were confirmed by two board-certified radiologists and reviewed by an interdisciplinary team of neurosurgeons to ensure accurate categorization.

### 2.2. Surgical Procedure

**Figure 1:**
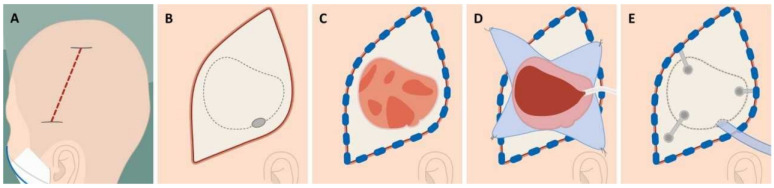
Surgical steps in acute subdural hematoma evacuation via craniotomy: (**A**) Scalp incision beginning above the ear and extending posteriorly over the temporoparietal region. (**B**) A single burr hole 4 cm above the external acoustic meatus. (**C**) Formation of a circa 6 × 6 cm bone flap. (**D**) Dural incision and evacuation of the subdural hematoma. (**E**) Placement of drainage to remove residual blood.

All craniotomies were performed by experienced neurosurgeons following a standardized surgical protocol to ensure consistency in the treatment approach [22]. The procedure involved the following steps, illustrated in Figure 1.

### 2.3. Imaging and Radiomic Analysis

CT imaging was performed preoperatively (within 24 h of injury) and postoperatively (within 24 h of surgery) following a standardized protocol. At our clinic, all patients undergoing craniotomy for aSDH routinely receive a native CT scan of the neurocranium the day after surgery, including both a 5 mm sequence and a 1 mm sequence. Imaging data were retrieved in DICOM format and analysed using 3D Slicer software (version 5.6.2). The radiomic analysis comprised several steps: Hematomas were manually segmented using the segmentation tools in 3D Slicer. After segmentation, a three-dimensional model of each hematoma was generated, and various radiomic features were extracted, including volume, surface area, Feret diameter, sphericity, flatness and elongation. These features were also measured on postoperative CT scans. The change in each feature (Δ) from preoperative to postoperative was subsequently calculated.

### 2.4. Statistical Analysis

Statistical analysis was conducted using IBM SPSS Statistics (version 29). The primary outcomes of interest were the 30-day outcomes, dichotomized as good (mRS ≤ 3) and poor (mRS > 3). The dichotomization was performed based on previously published RCTs [22,23,24]. Univariate analysis was performed using appropriate statistical methods tailored to the data distribution. Normality was assessed using standard tests, and depending on the results, Student’s *t*-test or the Mann–Whitney U test was applied for continuous variables, and the chi-squared test for categorical variables. To provide additional context, we conducted a sample size estimation based on data from our analysis. Assuming a moderate effect size (e.g., OR = 3.0 for Δ Surface Area as a predictor), an outcome distribution of approximately 31% (good outcome, mRS ≤ 3) vs. 69% (poor outcome, mRS > 3), two covariates, a significance level of 0.05, and 80% statistical power, a minimum sample size of 55 patients would be required for logistic regression.

Initially, univariate analysis was performed to evaluate the relationship between changes in radiomic features, clinical parameters, and laboratory parameters, including haemoglobin and haematocrit, in relation to the 30-day outcome.

Receiver Operating Characteristic (ROC) curve analysis was then conducted to determine the diagnostic accuracy of radiomic features. The area under the curve (AUC) was calculated for each feature, and the optimal cut-off value was identified.

Binary logistic regression was subsequently employed to assess the association between dichotomized radiomic pre- and postoperative features (based on ROC-derived cut-offs) for outcome prediction. Odds ratios (OR) were calculated along with 95% confidence intervals (CI) to quantify the risk associated with each feature. For age, a cut-off of <80 years was utilized based on a previously published study [4]. Similarly, the cut-off for the SOFA score was defined as <5 [25]. The preoperative hematoma volume cut-off value was set at 50 cm^3^ based on multiple studies [14,22,26,27,28,29].

Results are presented as mean ± standard deviation (SD) for continuous variables and as frequencies (percentages) for categorical variables. *p*-values < 0.05 were considered statistically significant. All statistical tests were two-tailed.

## 3. Results

### 3.1. Patient Characteristics

**Figure 2:**
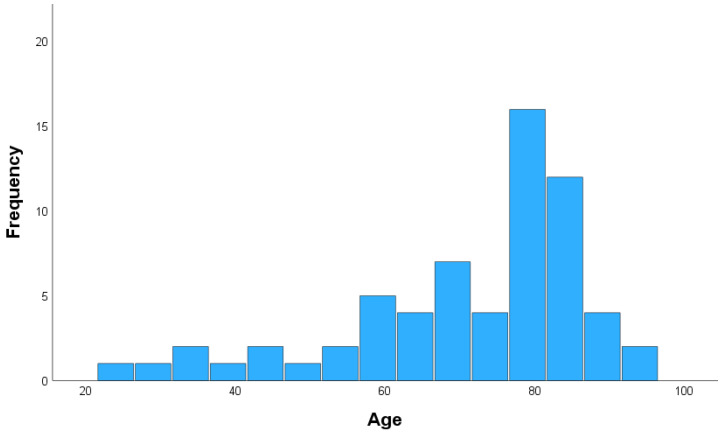
Age distribution.

The study included 64 patients who underwent craniotomy for aSDH evacuation. The mean age of the cohort was 70.81, with a higher proportion of male patients (36/64, 56.3%) (Figure 2).

### 3.2. Univariate Analysis

The pre- and postoperative radiomic features were analysed to assess their relationship with 30-day outcome. Table 1 summarizes these features across the overall cohort, comparing patients who achieved favourable outcomes with those who did not.

Patients with poor outcomes demonstrated a significantly larger mean change in surface area compared to those with good outcomes (37,602.56 ± 20,976.95 mm^2^ vs. 26,157.90 ± 17,328.26 mm^2^; *p* = 0.03). Additionally, the groups demonstrated significant differences in age (64.40 ± 18.42 years vs. 73.73 ± 14.20; *p* = 0.03) and presence of anisocoria on admission (1/20 vs. 12/44; *p*= 0.04). No significant differences were observed in cardiac comorbidities, history of anticoagulation, SOFA score or APACHE score.

Table 2 provides a detailed comparison of preoperative characteristics between patients with good and poor 30-day outcomes.

The surface area before surgery was significantly associated with poor outcomes (31,079.53 ± 1866.7 mm^2^ vs. 44,326.72 ± 22,045.30 mm^2^; *p* = 0.009), as was the Feret diameter (133.41 ± 28.17 mm vs. 150.01 ± 21.72 mm; *p* = 0.012). Elongation and sphericity did not demonstrate significant differences, nor did preoperative haemoglobin levels or haematocrit levels.

### 3.3. ROC Analysis and Cut-Off Values

**Figure 3:**
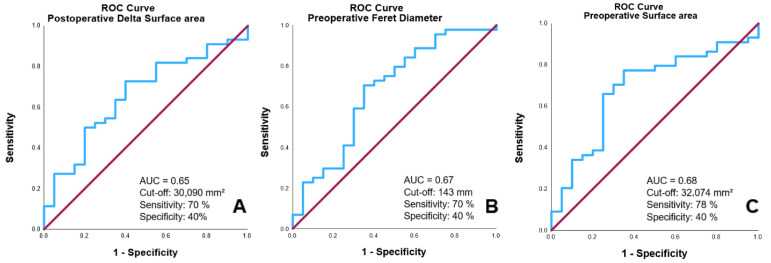
ROC Curves for predicting 30-day outcomes based on postoperative changes in postoperative Δ surface area (**A**), preoperative Feret diameter (**B**), and preoperative surface area (**C**).

To assess the predictive value of radiomic features related to 30-day outcomes, Receiver Operating Characteristic curve analyses were conducted:

Δ surface area showed an AUC of 0.65, with a sensitivity of 70% and a specificity of 40%. The ideal cut-off value is 30,090 mm^2^, as illustrated in Figure 3A.

Preoperative Feret diameter demonstrated an AUC of 0.67 with a sensitivity of 70% and a specificity of 40%. The ideal cut-off value is 143 mm, as shown in Figure 3B.

Preoperative surface area exhibited an AUC of 0.68, with a sensitivity of 78% and a specificity of 40%. The ideal cut-off value is 32,074 mm^2^, as depicted in Figure 3C.

### 3.4. Binary Logistic Regression

To evaluate the predictive factors associated with good and poor outcomes, we performed a binary logistic regression analysis. In this analysis, the pre- and postoperative radiomic features were dichotomized according to the cut-offs established in the previous ROC analysis. For age [4] and hematoma volume [26], cut-offs were determined based on previous studies on this topic. To ensure the absence of multicollinearity, a correlation analysis using Spearman’s rank correlation coefficient was performed. No variables exceeded the critical threshold of 0.7, with the highest correlation observed between Δ Surface Area and Feret Diameter (r = 0.664, *p* < 0.001).

A. Postoperative radiomic features, age, and pupillary response:

We included significant univariate factors such as change in surface area (Δ surface area), age, and pupillary response, as demonstrated in Table 3.

In the multivariate analysis, Δ surface area showed statistical significance, whereas pupillary response and age were not correlated with 30-day outcomes.

B. Preoperative and postoperative significant factors combined:

We included all significant univariate factors such as change in surface area (Δ surface area), Feret diameter, volume, pupillary response, and age.

Analysis of all factors combined indicated that preoperative Feret diameter was the strongest predictor of outcome, while the other variables were not statistically significant. For detailed information, see Table 4.

## 4. Discussion

This study explored the predictive value of radiomic features in patients with aSDH who underwent craniotomy. Utilizing advanced imaging software (3D Slicer), we assessed various radiomic characteristics of the hematomas, including surface area, volume, Feret diameter, sphericity, and flatness, before and after surgery. Our analysis identified change in surface area (Δ surface area) as the only independent postoperative radiomic predictor of 30-day outcomes. Additionally, preoperative Feret diameter emerged as the sole independent predictor of 30-day outcomes in binary logistic regression combining both pre- and postoperative statistically significant factors.

### 4.1. Radiomics

Radiomics utilizes advanced computer software to extract detailed quantitative information from medical images. By analysing geometric, texture-based, and intensity-related properties, radiomics provides a more comprehensive understanding of underlying hematoma characteristics. In oncology, radiomics has been widely employed to predict tumour progression and treatment outcomes [17]. Beyond oncology, radiomics is showing promising potential across various fields. For instance, in neurology, it may be useful in characterizing clots or guiding mechanical thrombectomy [30]. In neurosurgery, one study used 3D Slicer to assess hematoma irregularity indices for predicting hematoma expansion in intracerebral haemorrhage [31]. Another recent study applied radiomic analysis to acute intracerebral haemorrhage (ICH), identifying first-order energy as a significant radiomic feature predictive of survival at various time points, with AUC values exceeding 0.67 [32]. These radiomic features demonstrated prognostic utility comparable to established clinical scoring systems, such as ICH Score, further underscoring the potential of radiomics to guide patient management in acute neurological conditions.

The importance of radiomic analysis is further highlighted by recent studies demonstrating how this technology can identify specific radiomic features for clinically relevant outcomes. Wu et al. utilised radiomic features such as surface area-to-volume ratio and maximum axial diameter to develop a predictive model for recurrence risk in chronic subdural hematomas (cSDH) [33]. This model significantly improved predictive accuracy compared to traditional morphological features (AUC: 0.7998 vs. 0.7239). As highlighted by Gillies et al., integrating radiomic features with clinical and genomic data enhances precision medicine by identifying imaging biomarkers that guide tailored therapeutic strategies [34]. This underscores the potential of radiomics to improve outcomes in fields such as neurology and neurosurgery.

Radiomics enhances traditional CT analysis by extracting high-dimensional features that complement standard imaging biomarkers, enabling a more detailed understanding of morphological and textural properties. This integration has been shown to improve diagnostic and prognostic precision, bridging the gap between qualitative interpretation and quantitative decision support in clinical practice [35].

Clinical models, such as GCS, provide rapid assessments but often overlook the geometric complexity of hematomas. Radiological features primarily focus on basic volumetric measures, while AI-based models depend heavily on robust and standardized input data. Radiomic features, such as Feret diameter and preoperative surface area, quantitatively capture geometric complexity and could complement both clinical and AI-based models, significantly enhancing the accuracy and applicability of predictive frameworks [36,37]. The Feret diameter serves as a geometric parameter for evaluating the size of irregularly shaped lesions, such as acute subdural hematomas. It represents the maximum distance between any two points along the hematoma’s perimeter, offering insights into its morphological characteristics. An increased Feret diameter may indicate greater complexity of the hematoma, suggesting a more severe pathological state. Such hematomas may present a more insidious condition compared to those with a mathematically larger volume but a less complex shape (smaller Feret diameter).

A larger Δ surface area indicates more substantial hematoma removal, which may correlate with reduced hematoma–brain tissue contact, thereby limiting secondary neurotoxicity associated with hematoma degradation and indirectly improving patient outcomes [38]. Conversely, a minimal change in surface area might suggest inadequate evacuation, leaving residual hematoma that could contribute to ongoing neurotoxic processes and worse outcomes.

Radiomic analysis may provide valuable insights by identifying radiomic features associated with outcomes in aSDH. In our study, Δ surface area and preoperative Feret diameter emerged as significant predictors. Patients with a Δ surface area > 30,090 mm^2^ had poorer outcomes (OR = 6.66, *p* = 0.02), and preoperative Feret diameter was independently associated with recovery (*p* = 0.01). These parameters may assist in risk stratification and guide clinical decisions such as surgical timing and postoperative monitoring.

In related research, Hamou et al. identified internal hematoma architecture as a predictor of recurrence in chronic subdural hematomas (e.g., sedimented hematomas, 50% recurrence) [39]. Fang et al. demonstrated that machine learning models incorporating radiomic features achieved an AUC of 91.34% for predicting postoperative recurrence [40]. These findings highlight the potential of radiomics to enhance personalized care. However, while such studies highlight the promise of radiomics in acute cerebral haemorrhage, its application in aSDH remains largely unexplored. The lack of research in this area underscores the novelty of our study.

### 4.2. Additional Factors

Age is a well-established prognostic factor in TBI [41]. The mean age of our cohort was 70.81 years, consistent with existing data showing that aSDH predominantly affects the elderly [42]. As the global population ages, the incidence of aSDH increases. In the 1970s, the mean age of patients suffering from any form of TBI was around 50 years, rising to approximately 60 years in the 1980s [43]. By the 1990s and early 2010, this further increased to around 70 years, with recent data indicating that the mean age advanced towards 80 years [43]. Previous studies have reported mortality rates for elderly aSDH patients ranging from 35 to 67% [44,45], which corresponds with the findings in our cohort.

The majority of patients in our study were male (56.3%), reflecting trends observed in traumatic brain injury literature, where older men are more prone to experience head injuries [46,47]. Additionally, many of our patients presented with clinically relevant comorbidities, particularly concerning the use of blood thinners; 52% of patients had a positive history of anticoagulants, which is common among elderly patients with cardiovascular conditions in Germany [48]. Anticoagulant use is known to increase the risk of developing subdural hematoma [49] and is associated with poorer outcomes. However, in our study, blood thinners were not a major predictor of outcome [4]. This may be due to the strictly selected patient cohort, which only represents the subgroup of patients undergoing craniotomy. Furthermore, advancements in anticoagulation reversal agents and improvements in perioperative management might have significantly reduced the effect of blood thinners on general outcome. Given the widespread use of anticoagulants in this population, identifying cases of aSDH without anticoagulant involvement is challenging, potentially limiting our understanding of the broader spectrum of the condition and its outcomes [50].

Anisocoria has been linked to worse outcomes in previous studies [51]. In our cohort, we confirmed this finding in univariate analysis; however, the multivariate analysis showed that anisocoria is not an independent outcome predictor. This might be due to strict selection, where patients with more severe intracranial hypertension underwent primary decompressive craniectomy and are therefore not represented in our study. Furthermore, there might be retrospective bias due to preexisting conditions. Many patients were elderly, with multiple pre-existing conditions, which might have had a more dominant effect on outcomes than pupillary response. Additionally, the higher number of patients with isocoria and anisocoria in the poor outcome group (*n* = 44) compared to the good outcome group (*n* = 20) reflects the larger sample size of the former, which may contribute to the observed association in univariate analysis. This disparity, combined with the influence of other strong prognostic factors such as Glasgow Coma Scale scores and systemic comorbidities, likely diminishes the predictive value of anisocoria in multivariate analysis.

Preoperative haematocrit levels were not significant in the univariate analysis. However, haematocrit could still be a predictor as low levels may impair oxygen delivery, worsening ischemia, while high levels increase blood viscosity, reducing cerebral perfusion [52]. The multifactorial nature of brain injury and modern management strategies may overshadow the isolated role of haematocrit in predicting outcomes. Preoperative haemoglobin levels did not reach significance in the univariate analysis. Nonetheless, haemoglobin could still be a relevant predictor, as low levels impair oxygen delivery, potentially exacerbating cerebral ischemia [53]. Advances in perioperative management and transfusion strategies may have minimized the isolated impact of haemoglobin on clinical outcomes.

## 5. Limitations

There are several limitations to this study. First, the retrospective design limits the ability to establish causality between radiomic features and patient outcomes. Second, the study was conducted at a single institution. Another limitation of this study is the strict inclusion criteria, which focused exclusively on patients undergoing craniotomy. While this approach ensures a homogeneous cohort for radiomic analysis, it may introduce selection bias, as only 64 out of 178 initially identified patients were included. Patients who received alternative treatments, such as hemicraniectomy or conservative management, were excluded. This may limit the generalizability of our findings to broader patient populations. Third, while radiomic analysis has shown promise, the manual segmentation of hematomas introduces potential variability, despite attempts to standardize this process. Furthermore, while brain elasticity likely plays a role in patient recovery, it was not directly measured in this study. Instead, postoperative radiomic features, such as changes in surface area and Feret diameter, were used as indirect indicators of recovery dynamics. Future studies should consider automating this process to reduce bias. Further validation in larger, multicentre studies is needed to confirm these findings and ensure their broad applicability in clinical practice.

## 6. Conclusions

This study identifies radiomic features, specifically the change in hematoma surface area (Δ surface area) and Feret diameter, as significant predictors of 30-day outcomes in patients undergoing craniotomy for aSDH. Our findings indicate that changes in hematoma morphology, rather than size alone, play a critical role in determining patient outcomes. Radiomic analysis offers a quantitative and detailed assessment of these morphological changes, complementing traditional clinical and radiological metrics.

These features may enhance risk stratification and guide personalized follow-up strategies, such as optimizing the timing of postoperative imaging or tailoring the intensity of clinical monitoring. Future studies should focus on validating these findings in larger, multicentre cohorts and exploring their integration into clinical workflows and AI-based predictive models.

## Figures and Tables

**Table 1:** Postoperative radiomic features and their association with 30-day outcome, dichotomized as good (mRS ≤ 3) and poor (mRS > 3).

Variable	Overall Cohort	Good Outcome	Poor Outcome	*p*-Value
Δ Surface area (mm^2^)	34,026.10 ± 20,481.31	26,157.90 ± 17,328.26	37,602.56 ± 20,976.95	0.03
Postoperative/preoperative surface	0.19 ± 0.17	0.20 ± 0.18	0.19 ± 0.16	0.80
Elongation	1.82 ± 0.56	1.82 ± 0.56	1.81 ± 0.57	0.94
Flatness	3.33 ± 1.24	3.51 ± 1.12	3.24 ± 1.29	0.42
Sphericity	0.37 ± 0.15	0.70 ± 0.40	0.62 ± 0.35	0.42
Surface (mm^2^)	6242.08 ± 5368.61	4958.03 ± 4333.46	6825.73 ± 5728.39	0.19
Feret diameter (mm)	91.13 ± 26.33	83.37 ± 19.98	94.65 ± 28.25	0.11
Volume (cm³)	10.12 ± 14.84	14.11 ± 23.80	8.30 ± 7.84	0.14
APACHE Score	15.33 ± 6.41	14.40 ± 6.32	15.80 ± 6.48	0.42
SOFA Score	4.03 ± 2.96	3.80 ± 2.72	4.15 ± 3.09	0.66
Age (years)	70.81 ± 16.10	64.40 ± 18.42	73.73 ± 14.20	0.03
Anticoagulation	33 (Yes) vs. 31 (No)	8 (Yes) vs. 12 (No)	25 (Yes) vs. 19 (No)	0.21
Cardiac comorbidities	45 (Yes) vs. 19 (No)	12 (Yes) vs. 8 (No)	33 (Yes) vs. 11 (No)	0.22
Pupillary response	51 (Isocor) vs. 13 (Anisocor)	19 (Isocor) vs. 1 (Anisocor)	32 (Isocor) vs. 12 (Anisocor)	0.04

**Table 2:** Preoperative radiomic features and their association with good vs. poor 30-day outcomes.

Variable	Overall Cohort	Good Outcome (*n* = 20)	Poor Outcome (*n* = 44)	*p*-Value
Elongation	1.53 ± 0.29	1.44 ± 0.27	1.56 ± 0.29	0.12
Sphericity	0.27 ± 0.89	0.30 ± 0.9	0.25 ± 0.8	0.82
Surface area (mm^2^)	40,186.98 ± 21,796.96	31,079.53 ± 1866.7	44,326.72 ± 22,045.30	0.009
Feret diameter (mm)	144.83 ± 24.93	133.41 ± 28.17	150.01 ± 21.72	0.012
Volume (cm³)	124.97 ± 244.24	70.48 ± 42.85	106.75 ± 56.87	0.0001
Haemoglobin (mmol/l)	7.34 ± 1.20	6.97 ± 1.31	7.51 ± 1.12	0.09
Haematocrit	0.33 ± 0.05	0.32 ± 0.05	0.34 ± 0.05	0.14

**Table 3:** Multivariate logistic regression analysis of postoperative radiomic features, age, and pupillary response.

Variable	Odds Ratio (OR)	95% Confidence Interval (CI)	*p*-Value	Wald Statistic
Δ Surface Area	6.66	1.26–35.03	0.02	5.02
Pupillary response	4.75	0.51–44.23	0.17	1.87
Age	2.09	0.43–9.98	0.35	0.85

**Table 4:** Multivariate logistic regression analysis of all significant variables.

Variable	Odds Ratio (OR)	95% Confidence Interval (CI)	*p*-Value	Wald Statistic
Δ Surface Area	1.94	0.22–17.16	0.55	0.35
Pupillary response	8.72	0.91–83.19	0.06	3.54
Age	1.55	0.29–8.08	0.60	0.27
Feret diameter (preoperative)	8.94	1.58–50.49	0.01	6.16
Volume (preoperative)	0.85	0.12–5.76	0.87	0.02

## Data Availability

The data presented in this study are available on request from the corresponding author due to the sensitive nature of clinical data and adherence to ethical guidelines.

## Abbreviations

The following abbreviations are used in this manuscript:

AI	artificial intelligence
aSDH	acute subdural hematoma
AUC	area under the curve
cSDH	chronic subdural hematoma
CT	Computed tomography
GCS	Glasgow Coma Scale
ICH	intracerebral haemorrhage
OR	odds ratio
mRS	modified Rankin Scale
ROC	receiver operating characteristic
SD	standard deviation
TBI	traumatic brain injury
